# Oral Administration of Recombinant *Saccharomyces boulardii* Expressing Ovalbumin-CPE Fusion Protein Induces Antibody Response in Mice

**DOI:** 10.3389/fmicb.2018.00723

**Published:** 2018-04-13

**Authors:** Ghasem Bagherpour, Hosnie Ghasemi, Bahare Zand, Najmeh Zarei, Farzin Roohvand, Esmat M. Ardakani, Mohammad Azizi, Vahid Khalaj

**Affiliations:** ^1^Department of Medical Biotechnology, Pasteur Institute of Iran, Tehran, Iran; ^2^Department of Virology, Pasteur Institute of Iran, Tehran, Iran; ^3^Department of Molecular Medicine, Pasteur Institute of Iran, Tehran, Iran

**Keywords:** *Saccharomyces boulardii*, antigen delivery, recombinant ovalbumin, *Clostridium perfringens* enterotoxin, mucosal immunity

## Abstract

*Saccharomyces boulardii*, a subspecies of *Saccharomyces cerevisiae*, is a well-known eukaryotic probiotic with many benefits for human health. In the present study, a recombinant strain of *S. boulardii* was prepared to use as a potential oral vaccine delivery vehicle. In this sense, a ura3 auxotroph strain of *S. boulardii* CNCM I-745 (known as *S. cerevisiae* HANSEN CBS 5926, Yomogi^®^) was generated using CRISPR/Cas9 methodology. Then a gene construct encoding a highly immunogenic protein, ovalbumin (OVA), was prepared and transformed into the *ura3^-^ S. boulardii*. To facilitate the transport of the recombinant immunogen across the intestinal barrier, a claudin-targeting sequence from *Clostridium perfringens* enterotoxin (CPE) was added to the C-terminus of the expression cassette. The recombinant *S. boulardii* strain expressing the OVA-CPE fusion protein was then administered orally to a group of mice, and serum IgG and fecal IgA levels were evaluated by ELISA. Our results demonstrated that anti-OVA IgG in serum significantly increased in test group (*P* < 0.001) compared to control groups (receiving wild type *S. boulardii* or PBS), and the fecal IgA titer was significantly higher in test group (*P* < 0.05) than control groups. In parallel, a recombinant *S. boulardii* strain expressing the similar construct lacking C-terminal CPE was also administered orally. The result showed an increased level of serum IgG in group receiving yeasts expressing the CPE negative construct compared to control groups; however, the fecal IgA levels did not increase significantly. In conclusion, our findings indicated that the yeast *S. boulardii*, as a delivery vehicle with possible immunomodulatory effects, and c-CPE, as a targeting tag, synergistically assist to stimulate systemic and local immunity. This proposed recombinant *S. boulardii* system might be useful in the expression of other antigenic peptides, making it as a promising tool for oral delivery of vaccines or therapeutic proteins.

## Introduction

The yeast *Saccharomyces boulardii* is a known GRAS (generally regarded as safe) microorganism with the probiotic activity against a wide range of microbial pathogens in intestinal lumen (Czerucka et al., 2007). This probiotic yeast is often marketed in lyophilized form, called as *S. boulardii lyo*. The whole genome of *S. boulardii* has been sequenced, and the comparative genome analysis has been carried out (Khatri et al., 2017).

From a clinical point of view, the results of several randomized controlled trials in adult patients have frequently confirmed the significant positive effect of this probiotic in the treatment of acute and chronic diseases of intestine (Berni et al., 2011).

Based on several published data, the administration of *S. boulardii* in mice demonstrated immunomodulatory effects and resulted in increased levels of secretory IgA and serum IgG (Rodrigues et al., 2000; Qamar et al., 2001) as well as serum IgM (Stier and Bischoff, 2016). These immunological effects along with other probiotic features of *S. boulardii*, such as bile resistance, acid resistance, and the optimum growth temperature of 37°C, make this host as a potential vehicle for oral delivery of vaccines and other therapeutics into the lumen of intestine (Czerucka et al., 2007; Edwards-Ingram et al., 2007; Kelesidis and Pothoulakis, 2012). However, in a recent report, it has been shown that the immunoactivity of the probiotic yeast is limited in a healthy intestine as the majority of *S. boulardii* do not contact the gastrointestinal epithelium, and their uptake by Peyer’s patches (PPs) is infrequent (Hudson et al., 2016a). Hence, it is necessary to make arrangements for efficient expression and delivery of antigens or therapeutic proteins to intestinal epithelium by *S. boulardii*.

Chicken ovalbumin (OVA) has two antigenic epitopes (OVA 257–264 and OVA 323–339) that bind to MHC class I and class II (Johnsen and Elsayed, 1990; Rotzschke et al., 1991). For efficient delivery of the recombinant antigen to immune cells underlying intestinal epithelium, an epithelium targeting ligand, *Clostridium perfringens* enterotoxin (CPE), was chosen to target claudin. Claudins are a family of integral membrane proteins in tight junctions (TJs) with at least 24 members (Lu et al., 2013). Among them, claudin-3 and -4 can be targeted by CPE, which has been shown to act as a TJs modulator and to increase the adsorption of fused proteins by losing TJs in intestinal epithelium (Suzuki et al., 2012). Detailed studies, however, have demonstrated that the carboxy-terminal region of CPE (c-CPE, amino acids 194–319) binds claudins, while the NH_2_-terminal part of the molecule forms pores in the plasma membrane and induces cell death (Van Itallie et al., 2008). Here, the c-CPE sequence was fused C-terminally to OVA sequence, and the whole gene construct was expressed in *S. boulardii* CNCM I-745 as a probiotic yeast (Moré and Swidsinski, 2015; Stier and Bischoff, 2016; Kabbani et al., 2017). The recombinant *S. boulardii* was orally administered to C57BL/6 mice, and immunological assays were performed to evaluate possible immune responses.

## Materials and Methods

### Strains and Plasmids

*Saccharomyces boulardii* CNCM I-745 (Yomogi^®^) was used for the construction of ura3 auxotroph mutant, the expression of OVA fusion protein, and animal studies. *Saccharomyces cerevisiae* BY4742 was employed as a control strain in transformation and expression experiments. *Escherichia coli* strain Top 10F′ was used as a host for plasmid preparations. Yeasts strains were grown and kept in the YPD medium (1% yeast extract, 2% polypeptone, and 2% dextrose). Yeast nitrogen base medium (YNB; Sigma-Aldrich, United States) was prepared at a concentration of 0.67% and was supplemented with 2% glucose. For the selection of *ura3^-^* strains, YNB medium was supplemented with 0.1% 5-fluoroorotic acid (5-FOA, Sigma-Aldrich), and 10 mM uracil (YNB-FOA-U). The pGEM^®^-T Easy cloning vector (Promega, United States) was used as intermediate vector for cloning of various DNA fragments. The episomal yeast plasmid, pYES2 (Invitrogen, United States), containing *S. cerevisiae ura3* gene and the inducible Gal1 promoter was utilized as a control plasmid in transformation experiments as well as in preparation of expression cassettes. Plasmids carrying Cas9 (pTEF1p-Cas9-CYCt1_kanMX; Stovicek et al., 2015) and *ura3* gRNA (pTAJAK-98; Jakočiūnas et al., 2015) constructs were used in the preparation of *ura3* auxotroph yeast strain and were kindly provided by Irina Borodina (Technical University of Denmark).

Restriction endonucleases and T4 DNA ligase were obtained from Fermentas (Waltham, United States). Primers were synthesized by SinaClon BioScience (Tehran, Iran). All chemicals and reagents used were purchased from standard commercial sources.

### Preparation of ura3 Auxotroph Mutant of *S. boulardii*

A targeted gene inactivation method using CRISPR/Cas9 system was used to prepare the *ura3^-^* strain of *S. boulardii*. Plasmids pTEF1p-Cas9-CYCt1_kanMX and pTAJAK-98 were co-transformed into the wild-type *S. boulardii* using a standard electroporation method (Benatuil et al., 2010; Hudson et al., 2014). The electroporated cells were plated on YNB-FOA-U agar medium, and *ura3^-^* strains were recovered after 3 days at 30°C.

### Preparation of Different Ovalbumin Expression Constructs

**FIGURE 1:**
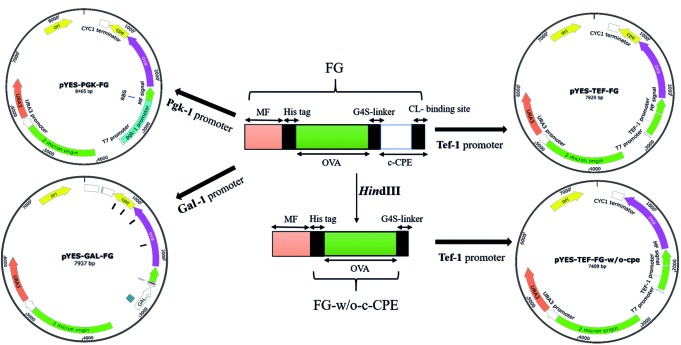
The schematic representation of constructed vectors. Three promoters, Tef-1, Pgk-1, and Gal-1, were used to drive the expression of the fusion gene (FG) by pYES-based episomal plasmids. Tef-1 promoter was selected as a strong promoter in *S. boulardii* (Yomogi^®^) and used for preparation of the CPE-less construct (pYES-TEF-FG-w/o-cpe).

The OVA amino acid sequence was retrieved from UniProt (accession number: P01012). An N-terminal secretion signal from *S. cerevisiae* alpha-mating factor (MF, pre-pro-region) was then added to OVA sequence. A 6×His tag sequence was also added to the N-terminus of OVA sequence immediately after MF sequence. For the targeted attachment of the expressed protein to TJs of intestinal epithelium, the c-terminal region of CPE (amino acids 194–319) was joined to OVA C-terminus using a (G4S)3 linker. The complete amino acid sequence of fusion protein (MF-6×His-OVA-G4S-CPE) was back-translated to DNA sequence, and the resulting sequence was codon optimized according to *S. cerevisiae* codon usage. To facilitate the cloning procedures, the sequence was further modified to contain *Sal*I and *Spe*I restriction sites at 5′ and 3′ ends, respectively. The sequence also carried two *Hin*dIII restriction sites at 5′ and 3′ ends of c-CPE for removing this fragment when required. The final fusion gene (FG) fragment (1667 bp) was synthesized commercially (Generay Biotech, Shanghai). To construct episomal expression cassettes, the synthetic FG was cloned into *Sal*I/*Spe*I site of pYES2 vector containing Gal1 promoter. Gal1 is one of the widely used inducible promoters in yeast expression vectors such as pYES and pESC series introduced by Invitrogen and Agilent, respectively. The resulting plasmid was called pYES2-Gal1-FG plasmid and used for subsequent construction of other episomal plasmids. For preparation of two new divergent expression cassettes, the Gal1 promoter in pYES2-FG was replaced by either Tef1 or Pgk1 promoters. Tef1 and Pgk1 are two strong constitutive promoters with a constant activity at various concentration of glucose (Partow et al., 2010). To prepare these vectors, both Tef1 and Pgk1 promoters were PCR amplified by High Fidelity Taq polymerase (TransGen Biotech Co., Ltd., United Kingdom). The Tef1 promoter (402 bp) was amplified from pPICZα plasmid (Thermo Fisher Scientific, United States) using specific primers Tef1F/Tef1R (**Table 1**). Likewise, the Pgk1 promoter (992 bp) was amplified from *S. cerevisiae* genome (GenBank accession number: CP020125.1, nucleotides 142078–143034) using Pgk1F/PgkR primers. These primer sets were designed in a way to add *Sac*I and *Sal*I restriction sites at 5′ and 3′ ends of PCR products, respectively. Each amplified promoter was separately cloned into the *Sac*I/*Sal*I site of pYES2-Gal1-FG, and the resulting plasmids were called as pYES2-Tef1-FG and pYES2-Pgk1-FG (**Figure 1**). For preparation of episomal construct lacking c-CPE fragment, the pYES2-Tef1-FG plasmid was digested by *Hin*dIII to cut out the c-CPE sequence and the resulting plasmid was religated to end up with pYES2-Tef1-FG-w/o-cpe.

**Table 1:** Primers used in this study.

Name	Primers
ova-F	AACTGCTGCTGACCAGGC
ova-R	TGAGAAATCTTCAAAGACTCGGCAG
Cpe-F	AGAGATTGAACTTGACCGACGC
cpe-R	CGTAATGATCTTTGACTCCGTCTCCC
Pgk-1F	atGAGCTCGGAAGTACCTTCAAAGAATG
Pgk-1R	tatGTCGACTTGTTTTATATTTGTTGTAAAAAG
Tef-1F	tatGAGCTCCACACACCATAGCTTCAAAATGTTTCTAC
Tef-1R	tatGTCGACCTTAGATTAGATTGCTATGCTTTC
ura3-F	TATGCATGCTCTAGTATAGTCTATAGTCCGTGG
ura3-R	TATGCATGCTTAGTTTTGCTGGCCGCATC

*The underlined sequences represent the restriction enzyme site in each primer.*

**FIGURE 2:**
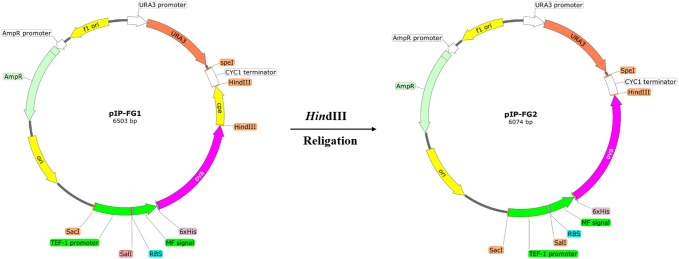
The schematic representation of the constructed Integrative plasmids. pIP-FG1 was contained Tef1 promoter, OVA-CPE gene sequence and ura3 selection marker. pIP-FG2 was prepared by *Hin*dIII digestion and subsequent relegation of pIP-FG1.

To prepare the integrative expression plasmid, the whole sequence of Tef1-MF-Ova-CPE was cut out from pYES2-Tef-FG vector using *Sac*I/*Spe*I digestion. This fragment was subsequently cloned into the respective sites of pGEM-T easy vector. To add the *ura3* selection marker to this integrative construct, the complete sequence of *ura3* gene was amplified from pYES2 vector using Ura3F/Ura3R primers (**Table 1**) and cloned into the *Sph*I site of the latter vector, yielding pIP-FG1. To make CPE-less expression cassette, the whole sequence of c-CPE fragment was released from pIP-FG1 by *Hin*dIII digestion, and the remaining vector was religated using T4 DNA ligase. This CPE-negative construct was called pIP-FG2 (**Figure 2**).

### Yeast Transformation

All yeast strains were transformed using a standard electroporation protocol as described before (Benatuil et al., 2010; Hudson et al., 2014). Briefly, an overnight culture of yeast was refreshed and grown in YPD broth medium to reach an OD600 of 1.6. Cells were washed with ice cold water and a buffer containing 1 M sorbitol and 1 mM CaCl_2_. Before electroporation, the cells were resuspended in 100 mM LiOAc/10 mM DTT solution and incubated at 30°C for 30 min. The cell suspension was then centrifuged, and the resulting pellet was resuspended in electroporation buffer (1 M sorbitol/1 mM CaCl_2_). Electroporation was performed in a volume of 400 μl mixture of cells and plasmid DNA (1 μg) using Gene Pulser Xcell^TM^ electroporation system (Bio-Rad Laboratories, United States). The transformation mixture was then spread on the selective medium, and positive transformants were recovered after 3 days incubation at 30°C.

### Analysis of Protein Expression

For the expression analysis of recombinant OVA (with or without CPE), yeast transformants were cultivated in 50 ml synthetic defined-casamino acid medium (SD-CAA: 5 g/l casamino acids, 20 g/l dextrose, 1.7 g/l yeast nitrogen base without ammonium sulfate/without amino acid, 10.2 g/l NaHPO_4_-7H_2_O, and 8.6 g/l NaH_2_PO_4_-H_2_O) and incubated at 37°C with shaking for 72 h. Afterward, the culture medium was collected by centrifugation and concentrated 50× using Amicon Ultra-15 Centrifugal Filtration system (Merck Millipore, Germany). The concentrated media (50 μl) were analyzed by sodium dodecyl sulfate-polyacrylamide gel electrophoresis (SDS-PAGE), and the expression of recombinant OVA was confirmed by Western blotting using a 1:500 dilution of unlabeled rabbit anti-OVA antibody (Millipore, Chemicon). A horseradish peroxidase (HRP)-conjugated goat anti-rabbit IgG (Razi Biotech Co., Iran) was used as the secondary antibody (1:2000 diluted) to detect the immunoreactive bands, followed by visualization with a chemiluminescence reagent (Amersham, United States). For the quantification of secreted recombinant protein, a His-Tag Protein assay Kit (Cell Biolabs, Inc., United States) based on competitive ELISA was used according to the protocol provided by the manufacturer.

### Animal Study

**FIGURE 3:**
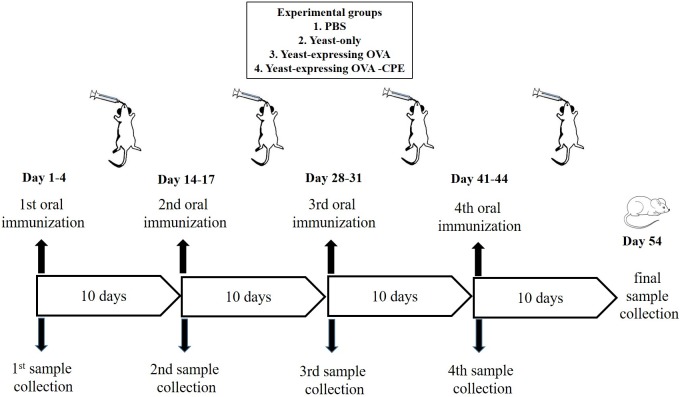
Schematic representation of oral immunization protocol using recombinant *S. boulardii* (Yomogi^®^).

Female C57BL/6 mice (6–8 weeks old) were provided by the animal facility of Pasteur Institute of Iran. The mice were housed at 23 ± 1°C with a 12-h light/dark cycle and were allowed free access to standard rodent chow and water. After their arrival, the mice were permitted to adapt to their environment for at least 1 week before the experiments. The study was approved by the Ethics Committee of Pasteur Institute of Iran and conforms to the European Communities Council Directive of 24 November 1986 (86/609/EEC). Oral immunizations were performed after an overnight fasting of the mice (water was provided *ad libitum*). An oral dose of 2 × 10^9^ yeast cell/mouse was administered using gavage for four successive days, followed by a boost immunization with the same amount of yeasts four times with a 10-day interval (**Figure 3**; Shin et al., 2007). Previous studies have shown that *S. boulardii* reaches to a steady state concentration in colon after 3 days of oral administration and accordingly our feeding was continued for 4 days in each oral immunization step (Blehaut et al., 1989). In this oral vaccination study, the experimental groups of three mice were arranged as follows: group A (PBS), group B (yeast only), group C (yeast expressing CPE negative OVA), and group D (yeast expressing OVA-CPE).

### Sample Preparation and Immunological Assays

Sample preparation was based on the previous works (Dion et al., 2004; Kakutani et al., 2010). Serum and fecal samples were collected 10 days after the last immunization. Fecal pellets (100 mg) were suspended in 1 ml of PBS buffer (pH 7.6) and in complete protease inhibitor cocktail tablets from Roche (Switzerland; one mini-tablet, EDTA-free, per 10 ml buffer) by vortexing for 10 min. Then samples were centrifuged at 3000 × *g* for 10 min, and the final supernatants were used as fecal extracts. The titer of OVA-specific antibody in serum and extracts were determined by ELISA. For ELISA, a Maxisorb multiwall plate (Nunc, Roskilde, Denmark) was coated with chicken OVA (100 ng/well). The plates were washed with PBS containing 0.05% Tween 20 (PBS-T) and blocked with 200 μl blocking buffer (PBS-T containing 2% skim milk) at 37°C for 1 h. Subsequently, 10-fold serial dilutions of these samples were added to the immunoplate, followed by the addition of HRP-conjugated anti-mouse IgG and IgA. The OVA-specific antibodies were detected using TMB peroxide substrate. Absorbance of reactions was read at 450 nm. All ELISA tests were performed in duplicates.

### Statistical Analysis

Statistical analysis of the total IgG and IgA levels were performed with Prism version 6.07 (Graph Pad Software, La Jolla, CA, United States). Experimental and control groups differences were analyzed by one-way analysis of variance (ANOVA), and the *P*-values of <0.05 were considered as statistically significant.

## Results

### Generation of *S. boulardii* (Yomogi^®^) ura3 Auxotroph Strain

**FIGURE 4:**
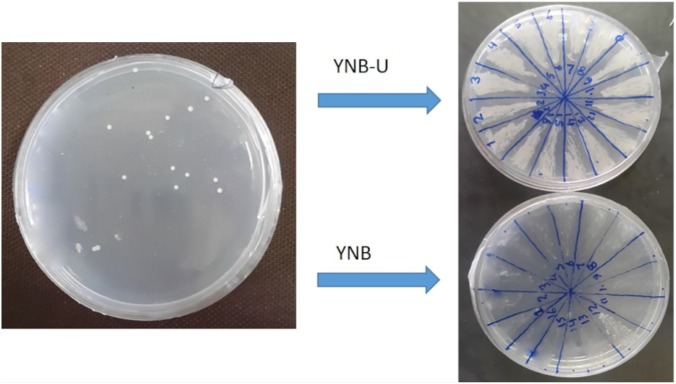
Isolation of ura3 auxotroph strains of *S. boulardii*. The auxotroph mutants were able to grow in YNB-U medium but not in YNB.

To generate a stable uracil auxotroph strain, the deletion of *ura3* gene was mediated by CRISPR/Cas9 system. Co-transforming of pTEF1p-Cas9-CYCt1_kanMX and pTAJAK-98 plasmids into *S. boulardii* resulted in several transformants on YNB-FOA-U agar medium. To confirm the inactivation of *ura3* gene, these transformants were grown on YNB and YNB-U media (**Figure 4**); the ura3 auxotrophs are not able to grow in YNB lacking uracil but grow normally in YNB-U. After primary confirmation, one colony was selected and analyzed further. This selected colony was transformed with pYES2 vector carrying *ura3* gene, and the resulting transformants were recovered on YNB medium. The complementation of *ura3^-^* mutant of *S. boulardii* by pYES2 confirmed the successful deactivation of *ura3* gene in this strain by CRISPR/Cas9 method. The growth phenotype of this ura3 auxotroph strain was compared with wild type, and the results did not detect any significant difference between the wild-type and the mutant strain (data not shown).

### Expression Analysis of OVA-CPE Fusion Protein in *S. cerevisiae* and *S. boulardii* CNCM I-745

**FIGURE 5:**
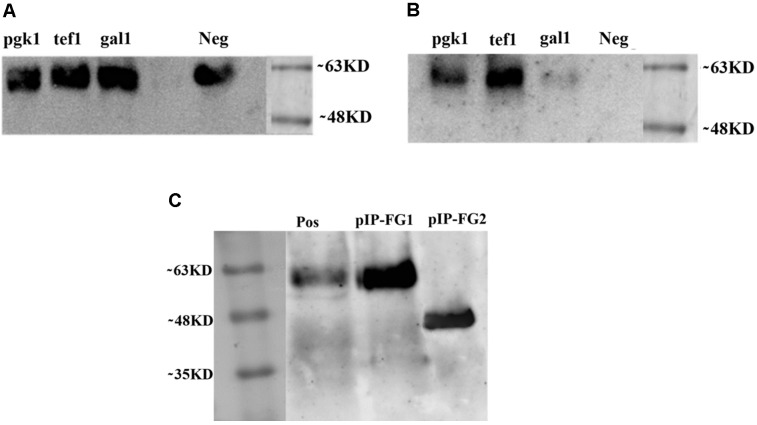
Western blot analysis on culture supernatant of different recombinant yeasts. Constitutive promoters, Pgk-1 and Tef-1, and inducible promoter, Gal-1 were used in preparation of constructs. **(A)** Expression of OVA-CPE (∼60 kD) by *S cerevisiae* BY 4742 strain using episomal constitutive and inducible promoters. **(B)** Expression of same constructs in *S. boulardii* (Yomogi^®^) strain. **(C)** Expression of integrative constructs, pIP-FG1 and pIP-FG2 in *S. boulardii.* The smaller size of CPE-less construct (∼45 kD) is shown.

To validate our constructed vectors (pYES2-Gal1-FG, pYES2-Tef-FG, and pYES2-Pgk-FG) and select the most suitable promoter, all the pYES2-based vectors were individually transformed into *S. cerevisiae* BY4742, and the expression pattern was analyzed as described earlier. SD-CAA was used as expression medium for all constructs except for pYES2-Gal1-FG containing Gal1 promoter in which all transformants were cultivated in the presence of galactose rather than glucose. As shown in **Figure 5A**, in *S. cerevisiae* BY4742, all the episomal plasmids successfully expressed OVA-CPE fusion protein (∼60 kD). However, we could not detect any expression by pYES2-Gal1-FG construct in *S. boulardii* (**Figure 5B**). Based on quantitative analysis of His-Tag OVA, the yield of expressed protein by pYES2-Tef-FG construct was 4.7 μg/4.3 × 10^9^cells/ml. The amount of secreted protein by pYES2-Pgk-FG construct in similar cultivation mode was 2.1/4.3 × 10^9^cells/ml, indicating more efficiency of Tef1 promoter. The Western blot analysis results also showed a stronger band of the target protein when the Tef1 promoter was used. Hence, the Tef1 promoter was chosen for preparation of integrative construct, pIP-FG1. As it is indicated in **Figure 5C**, the Western blot analysis of culture supernatant from pIP-FG1 and pIP-FG2 transformants of *S. boulardii* confirmed the successful expression and the secretion of OVA-CPE and CPE negative OVA (∼45 kD) proteins into the culture medium.

### Immunological Assay

**FIGURE 6:**
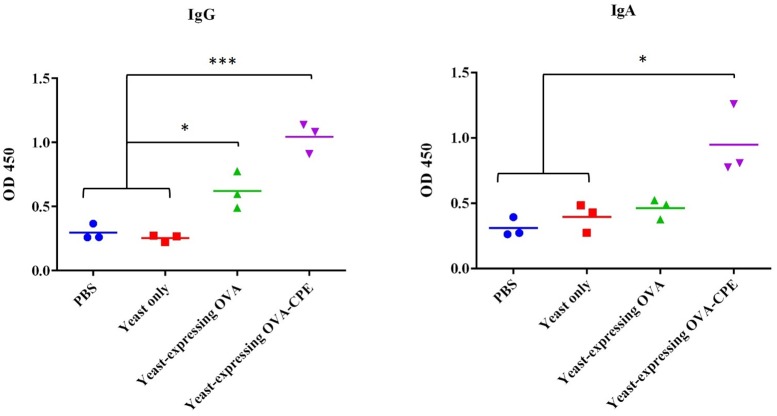
Evaluation of antibody response in mice. Systemic IgG and local IgA immune assay was done for serum and fecal samples, respectively. Serum IgG in both yeast expressing OVA-CPE (pIP-FG1 construct) and yeast expressing OVA (pIP-FG2 construct) groups was significantly higher than control groups (PBS and yeast only). Fecal IgA was higher in pIP-FG1 group in comparison to pIP-FG2 and the control groups (^∗^*P* < 0.05, ^∗∗∗^*P* < 0.001).

To find whether the oral administration of the recombinant *S. boulardii* is able to stimulate any anti-OVA-specific IgG and IgA responses, both serum anti-OVA IgG and fecal anti-OVA IgA were measured by ELISA. The sera and feces of every mouse were collected 10 days after each oral intubation and analyzed. However, the level of antibodies did not rise, until 10 days after the last intubation. Those C57BL/6 mice that were fed either with yeast expressing OVA-CPE (pIP-FG1 construct transformant, *P* < 0.001) or with OVA alone (pIP-FG2 construct transformant, *P* < 0.05) showed a significant IgG response compared to the control groups (wild-type *S. boulardii*-fed and PBS-fed groups). The measurement of fecal anti-OVA IgA antibody revealed that in the OVA-CPE treated group (*P* < 0.05), the IgA titers were significantly higher than that of the control group, however, there was no meaningful difference between IgA titers in mice that were fed yeast expressing OVA and controls (**Figure 6**).

## Discussion

Recently, attention has been focused on *S. boulardii* as a probiotic with the potential of engineering to produce a wide range of recombinant therapeutics (Hamedi et al., 2013; Hudson et al., 2014). Previous studies have highlighted the role of this yeast in the improvement of intestinal inflammatory or infective conditions. Three different mechanisms of action have been proposed for *S. boulardii*, including luminal action, trophic action, and mucosal-anti-inflammatory signaling effects (McFarland, 2010).

A recent report by Hudson et al. (2016a) has suggested that the therapeutic effect of *S. boulardii* is mainly attributed to the local activities of this yeast inside the lumen, including interfering with pathogen attachment or releasing antimicrobial toxins, but are not immune-mediated. Furthermore, in the same report, it has been found that *S. boulardii* does not induce any antibody response against its own antigens. The latter finding is an advantage in using this yeast for the repeated administration of various target antigens.

Mucosal vaccination is a simple and painless vaccination method that activates both the systemic and mucosal immune responses (Holmgren and Svennerholm, 2012). The oral administration route is particularly attractive as it is non-invasive and shows a good patient compliance (Zhu and Berzofsky, 2013). However, a major challenge for antigen proteins is to maintain their integrity while passing through the harsh environment of stomach and intestine. As a probiotic yeast, *S. boulardii* is able to survive in the acidic condition of stomach and to tolerate bile acids, and it is also capable of maintaining heterologous protein expression inside the intestine (Hudson et al., 2014). Hence, it can serve as a suitable carrier for the safe delivery of antigenic peptides to the intestinal lumen. Despite the above facts, there are some limiting factors such as the presence of intestinal protective mucus layer that hampers the efficient access of *S. boulardii* to immune system effectors (Edwards-Ingram et al., 2007). The gut-associated lymphoid tissue (GALT) is involved in antigen-specific mucosal immune responses in the gastrointestinal tract (Iijima et al., 2001). The initial uptake of antigens occurs in PPs of GALT, and few studies have demonstrated the infrequent presence of live *S. boulardii* in PPs after oral administration (Elmore, 2006; Hudson et al., 2014, 2016b). Moreover, the apical and basal TJs between intestinal epithelial cells act as a barrier, preventing macromolecules to access the paracellular spaces (Mayer, 2003).

Here, we hypothesized that the immune system can be stimulated if a target antigen released into the intestinal lumen by an engineered *S. boulardii*. Our target protein (OVA) was armed with c-CPE sequence to allow more penetration into the epithelium and more access to immune cells.

The first step in the construction of engineered *S. boulardii* was the generation of a ura3 auxotroph strain to use as a host for the transformation of various expression cassettes. Various methods, including classical UV mutagenesis or molecular tools such as Cre-loxP system, have been used for disruption of *ura3* in *S. boulardii* (Hamedi et al., 2013; Hudson et al., 2014; Wang et al., 2015). Here, we applied the CRISPR/Cas9 gene deletion method to knock out the *ura3* gene activity. CRISPR/cas9 methodology is a simple, reliable, and cheap method of genome editing. It promotes the sequence-specific dsDNA breaks, followed by repair via non-homologous end joining or homologous recombination. This method is particularly efficient when the host contains more than one copy of the target gene (Wilkinson and Wiedenheft, 2014). Zhang et al. (2014) have constructed a quadruple auxotrophic mutant of *S. cerevisiae* using RNA-guided Cas9 nuclease. Recently, Liu et al. (2016) have used CRISPR/Cas9 gene editing system to create a triple auxotroph of *S. boulardii* used in metabolic engineering.

To drive the transcription of expression cassettes, three different promoters, GAL-1, transcriptional elongation factor EF-1α (Tef-1), and phosphoglycerate kinase (Pgk-1) were used. Our expression experiments showed that *S. cerevisiae* (BY4742) but not *S. boulardii* CNCM I-745 (Yomogi^®^) is able to express OVA when Gal-1 promoter is used. This finding is in agreement with other studies that demonstrated the lack of galactose utilization by *S. boulardii* (Mitterdorfer et al., 2001). Using two other promoters, Tef-1 and Pgk-1, our target protein was successfully expressed and secreted into the culture medium. These promoters have been examined before, and it has been shown that Tef-1 promoter had stronger activity in both glucose-consuming and glucose-exhausted phases compared to Pgk-1 promoter (Partow et al., 2010). Similarly, in our experiments, Tef-1 promoter resulted in a stronger expression pattern similar to Partow et al.’s (2010) report.

In our experiments, the genetically engineered *S. boulardii* was orally administered, and immunological assays confirmed both IgA and IgG responses to OVA. Although both OVA (group C) and OVA-CPE (group D) expressing yeasts could induce IgG response in mice, the highest antibody response was observed in group D (*P* < 0.001). An explanation could be the presence of CPE partner that targets CL-4 at TJs. CL-4 is highly expressed in follicle-associated epithelium (FAE) of PPs in GALTs (Tamagawa et al., 2003), and its targeting by CPE may facilitate the access of CPE-fused antigen to initiators of immune response. Targeting of claudins, especially CL-4, by CPE-fused OVA and its efficiency in mice immunization have been already proved by Suzuki et al. (2012). Regarding the local immune response, IgA was detected at higher levels in group D with a significant difference (*P* < 0.05) compared to other groups. In group C although IgA was induced against OVA, the difference was not significant compared to the control group. The latter observation was in agreement with a previous report on weak IgA response induced by CPE negative OVA in mucosal immunization (Suzuki et al., 2012).

Taken together, the results of the present study indicate the use of *S. boulardii* as a probiotic with the ability of delivering immunogenic or therapeutic proteins to intestinal lumen *via* oral administration. Similar studies using different engineered yeasts have also shown promising results, leading to the description of “Whole Yeast Vaccine” as a new platform for vaccine development (Shin et al., 2007; Wang et al., 2016; Roohvand et al., 2017). Although we did not investigate the presence of intact antigens in the intestinal lumen, the induction of both serum IgG and fecal IgA responses indicate the release and accessibility of the recombinant antigen to the immune effector cells. In this study, we have used c-CPE for tight junction targeting and augmentation of intestinal absorption of foreign antigens. However, this process can be further improved by addition of other ligands such as transcytotic peptides (TP) (Kang et al., 2008; Lee et al., 2015), which have been recommended for intestinal absorption of large molecules such as therapeutic peptides.

## Author Contributions

GB performed the experiments and wrote the first draft of the manuscript. HG helped in protein expression experiments. BZ helped in gene construct preparations. NZ helped in yeast transformation and the related set ups. FR helped in animal study design and immunological assays. EA supervised animal handling and immunization steps. MA helped in gene constructs’ design. VK designed and coordinated the study, supervised all experimental steps, and revised and finalized the manuscript.

## Conflict of Interest Statement

The authors declare that the research was conducted in the absence of any commercial or financial relationships that could be construed as a potential conflict of interest.
